# PRISMA for Abstracts: Reporting Systematic Reviews in Journal and Conference Abstracts

**DOI:** 10.1371/journal.pmed.1001419

**Published:** 2013-04-09

**Authors:** Elaine M. Beller, Paul P. Glasziou, Douglas G. Altman, Sally Hopewell, Hilda Bastian, Iain Chalmers, Peter C. Gøtzsche, Toby Lasserson, David Tovey, for the PRISMA for Abstracts Group

**Affiliations:** 1 Centre for Research in Evidence-Based Practice, Bond University, Gold Coast, Australia; 2 Centre for Statistics in Medicine, University of Oxford, Oxford, United Kingdom; 3 National Center for Biotechnology Information, National Library of Medicine, Washington DC, United States of America; 4 James Lind Initiative, Oxford, United Kingdom; 5 Nordic Cochrane Centre, Copenhagen, Denmark; 6 Cochrane Editorial Unit, London, United Kingdom; 7 INSERM, Paris, France

## Abstract

Elaine Beller and colleagues from the PRISMA for Abstracts group provide a reporting guidelines for reporting abstracts of systematic reviews in journals and at conferences.

**Summary Points**

- The abstract of a systematic review should provide a structured summary that enables a quick assessment of the review's validity and applicability, and easy identification in electronic searching.
- Despite published guidance on writing the abstract in the PRISMA Statement guiding the reporting of systematic reviews in general and elsewhere, evaluations show that reporting of systematic reviews in journal and conference abstracts is poor.
- We developed consensus-based reporting guidelines as an extension to the PRISMA Statement on good reporting of systematic reviews and meta-analyses in abstracts.
- The PRISMA for Abstracts checklist gives authors a framework for condensing their systematic review into the essentials for an abstract that will meet the needs of many readers.

## Introduction

When readers screen the title of an article, and parts of its abstract, they try to determine whether or not to devote their scarce time to reading on. Some may be screening literature to identify the articles that are systematic reviews. Thus, the main function of an abstract of a systematic review should be to signal its systematic methodology. For most readers, the findings described in the abstract will also be key, either as the sole part of an article that will be read, or to determine whether reading the full text is required. Abstracts of systematic reviews are very important, as some readers cannot access the full paper, such that abstracts may be the only option for gleaning research results. This can be because of a pay wall, low Internet download capacity, or if the full article is only available in a language not understood by the reader. Readers in countries where English is not the primary language may have access to an abstract translated to their own language, but not to a translated full text. Conversely, a large proportion of systematic reviews are published by health technology agencies in non-English speaking countries [1], many of which provide only the abstract in English.

The predominance of the abstract in biomedical literature use is clear. Within queries to PubMed, most readers look only at titles; only half of searches result in any clicks on content [2]. The average number of titles clicked on to obtain the abstract or full text, even after retrieving several searches in a row, is less than five. Of those clicks, abstracts will be represented about 2.5 times more often than full texts of articles [2]. Even people going straight to a PDF or full text are likely to start, and perhaps end, with reading the abstract. The frequency of viewing full texts is somewhat higher among people searching the Cochrane Database of Systematic Reviews [3], but the same pattern is clear. After the title, the abstract is the most read part of a biomedical article.

Abstracts can be useful for screening by study type [4]; facilitating quick assessment of validity [4],[5]; enabling efficient perusal of electronic search results [4],[6]; clarifying to which patients and settings the results apply [4],[5]; providing readers and peer reviewers with explicit summaries of results [5]; facilitating the pre-publication peer review process [7]; and increasing precision of computerised searches [6],[7].

Structured abstracts were introduced in the medical literature about 25 years ago [4]–[6]. They provide readers with a series of headings, generally about the purpose, methods, results, and conclusions of the report, and have been adopted by many journals and conferences. They act as a prompt to the writer to give more complete information, and facilitate the finding of information by the reader.

Despite the adoption of structured abstracts, studies of the quality of abstracts of clinical trials have demonstrated that improvement is needed [8],[9], and a study of systematic review abstracts demonstrated that the direction of the effect or association could not be determined in one in four abstracts from the general and specialty medical literature [10]. The PRISMA Statement [11] gives some guidance for abstracts, closely linked to commonly used headings in structured abstracts. After observing that the quality of abstracts of systematic reviews is still poor [10], we decided to develop an extension to the PRISMA Statement to provide guidance on writing abstracts for systematic reviews. We also wanted to provide a checklist enabling the items suggested to fit into any set of headings mandated by a journal or conference submission.

## Methods for Development of the Checklist

We established a steering committee (EMB, PPG, SH, DGA). In collaboration with the steering group of the PRISMA Statement [11], we used the Statement to inform our selection of potential items for the checklist of essential items that authors should consider when reporting the primary results of a systematic review in a journal or conference abstract. The committee generated a list of items from PRISMA and other sources of guidance and information on structured abstracts and abstract composition and reporting [7],[11],[12], which were found using a thorough search of the literature.

In preparation for a consensus meeting, we used a modified Delphi consensus survey method [13] to select and reduce the number of possible checklist items. Each item was rated by survey participants as “omit”, “possible”, “desirable”, or “essential” to include in the final checklist. From the first round of the survey, the ranked items were divided into three lists for the second round. The first list contained the items with the highest rankings, and participants for the second round were instructed that these would be contained in the checklist unless they received low rankings in the second round. The second list contained the items with moderate rankings, and participants were instructed that these items were likely to be removed from the checklist unless they received high rankings in the second round. The third list contained the items with low rankings, and participants were instructed that these items would be removed unless they received very high rankings in the second round.

For the third round of the Delphi survey, a draft checklist was presented, which included only the items ranked highest in rounds one and two. The five next highest-ranked items were then presented, giving participants an opportunity to choose to include these in the checklist as well.

One hundred and forty-seven participants, who were authors of research on abstracts, established authors of systematic reviews, methodologists or statisticians related to systematic reviews, and journal editors, were invited by email to complete the three rounds of the web-based survey. The response rate was 68% (*n* = 100) for the first round. Only those who completed round one were invited to participate in rounds two and three. The response rate for round two was 80% (*n* = 80) and for round three 88% (*n* = 88).

The results of the survey were reported at a two-day consensus-style meeting on 13–14 October 2011, in Oxford, United Kingdom. Fifteen invited experts attended, most of whom had participated in the survey. The meeting began with a review of the literature about abstract structure and content, followed by a review of the checklist items as proposed by the survey respondents. Meeting participants discussed the items and agreed whether they should be included and how each item should be worded.

Following the meeting, the checklist was distributed to the participants to ensure it reflected the decisions made. This explanatory document was drafted and circulated through several iterations among members of the writing subcommittee who had all participated in the meeting. We developed this document using the template for the PRISMA Statement [11], which in turn was based on the methods of the CONSORT Group [14],[15].

## Scope of PRISMA for Abstracts

The PRISMA for Abstracts checklist focuses on truthful representation of a systematic review in an abstract. We developed the checklist to help authors report all types of systematic reviews, but recognise that the emphasis is on systematic reviews of evaluations of interventions where one or more meta-analyses are conducted. Authors who address questions on aetiology, diagnostic test accuracy, or prognosis may need to modify items or include other items in their abstract to reflect the essentials of the full report.

## The PRISMA for Abstracts Checklist

The checklist is shown in Table 1. An explanation for each item is given below. Citations for the examples of good reporting are in Table 2.

**Table 1:** The PRISMA for Abstracts Checklist.

**TITLE**	
1. Title:	Identify the report as a systematic review, meta-analysis, or both.
**BACKGROUND**	
2. Objectives:	The research question including components such as participants, interventions, comparators, and outcomes.
**METHODS**	
3. Eligibility criteria:	Study and report characteristics used as criteria for inclusion.
4. Information sources:	Key databases searched and search dates.
5. Risk of bias:	Methods of assessing risk of bias.
**RESULTS**	
6. Included studies:	Number and type of included studies and participants and relevant characteristics of studies.
7. Synthesis of results:	Results for main outcomes (benefits and harms), preferably indicating the number of studies and participants for each. If meta-analysis was done, include summary measures and confidence intervals.
8. Description of the effect:	Direction of the effect (i.e., which group is favoured) and size of the effect in terms meaningful to clinicians and patients.
**DISCUSSION**	
9. Strengths and Limitations of evidence:	Brief summary of strengths and limitations of evidence (e.g., inconsistency, imprecision, indirectness, or risk of bias, other supporting or conflicting evidence).
10. Interpretation:	General interpretation of the results and important implications.
**OTHER**	
11. Funding:	Primary source of funding for the review.
12. Registration:	Registration number and registry name.

**Table 2:** List of references used as examples.

Example Number	Citation
1a.	Branco BC, Barmparas G, Schuriger B, Inaba K, et al. (2010) Systematic review and meta-analysis of the diagnostic and therapeutic role of water-soluble contrast agent in adhesive small bowel obstruction. British Journal of Surgery 97: 470–478.
1b.	Agarwal R, Aggarwal AN, Gupta D, Jindal SK (2010) Inhaled corticosteroids vs placebo for preventing COPD exacerbations: a systematic review and metaregression of randomized controlled trials. Chest 137(2): 318–325.
2a.	Non-Small Cell Lung Cancer Collaborative Group (2010) Chemotherapy and supportive care versus supportive care alone for advanced non-small cell lung cancer. Cochrane Database of Systematic Reviews. Issue 5, Art. No. CD007309. DOI:10.1002/14651858.CD007309.pub2.
2b.	Kemp J, Armstrong L, Wan Y, Alagappan VKT, Ohlssen D, Pascoe S (2011) Safety of formoterol in adults and children with asthma: a meta-analysis. Annals of Asthma, Allergy and Immunology 107(1): 71–78.
2c.	Zhang Z, Ni H (2011) C-reactive protein as a predictor of mortality in critically ill patients: a meta-analysis and systematic review. Anaesthesia and Intensive Care 39(5): 854–861.
3a.	Chroinin M, Lasserson TJ, Greenstone I, Ducharme FM (2009) Addition of long-acting beta-agonists to inhaled corticosteroids for chronic asthma in children. Cochrane Database of Systematic Reviews Issue 3. Art. No. CD007949. DOI:10.1002/14651858.CD007949.
3b.	Clarke MJ, Hopewell S, Juszczak E, Eisinga A, Kjeldstrøm M (2006) Compression stockings for preventing deep vein thrombosis in airline passengers. Cochrane Database of Systematic Reviews. Issue 2, Art. No. CD004002. DOI:10.1002/14651858.CD004002.pub2.
3c.	Steinhart AH, Ewe K, Griffiths AM, Modigliani R, Thomsen OO (2003) Corticosteroids for maintenance of remission in Crohn's disease. Cochrane Database of Systematic Reviews Issue 4. Art. No. CD000301. DOI:10.1002/14651858.CD000301.
3d.	Trinh NH, Hoblyn J, Mohanty S, Yaffe K (2003) Efficacy of cholinesterase inhibitors in the treatment of neuropsychiatric symptoms and functional impairment in Alzheimer disease: a meta-analysis. Journal of the American Medical Association 289(2): 210–216.
4a.	Bonuck KA, Freeman K, Henderson J (2009) Growth and growth biomarker changes after adenotonsillectomy: systematic review and meta-analysis. Archives of Disease in Childhood 94: 83–91. DOI:10.1136/adc.2008.141192.
4b.	Loke YK, Singh S, Furberg CD (2009) Long-term use of thiazolidinediones and fractures in type 2 diabetes: a meta-analysis. CMAJ 180(1): 32–39.
5a.	Cho S-H, Lee H, Ernst E (2010) Acupuncture for pain relief in labour: a systematic review and meta-analysis. BJOG 117: 907–920.
6a.	Huss A, Scott P, Stuck AE, Trotter C, Egger M (2009) Efficacy of pneumococcal vaccination in adults: a meta-analysis. CMAJ 180(1): 48–58.
6b.	Alexander LD, Gilman DRD, Brown DR, Brown JL, Houghton PE (2010) Exposure to low amounts of ultrasound energy does not improve soft tissue shoulder pathology: a systematic review. Physical Therapy 90(1): 14–25.
7a.	Al-Majed NS, McAlister FA, Bakal JA, Ezekowitz JA (2011) Meta-analysis: cardiac resynchronization therapy for patients with less symptomatic heart failure. Annals of Internal Medicine 154: 401–412.
7b.	Wilson A, Gallos ID, Plana N, Lissauer D, Khan KS, Zamora J et al (2011) Effectiveness of strategies incorporating training and support of traditional birth attendants on perinatal and maternal mortality: meta-analysis. BMJ 343: d7102. DOI: 10.1136/bmj.d7102
7c.	Gillies M, Palmateer N, Hutchinson S, Ahmed S, Taylor A, Goldberg D (2010) The provision of non-needle/syringe drug injecting paraphernalia in the primary prevention of HCV among IDU: a systematic review. BMC Public Health 10: 721.
8a.	Jolly SS, Amlani S, Hamon M, Yusuf S, Mehta SR (2009) Radial versus femoral access for coronary angiography or intervention and the impact on major bleeding and ischemic events: a systematic review and meta-analysis of randomized trials. American Heart Journal 157(1): 132–140.
8b.	Lam LL, Cameron PA, Schneider HG, Abramson MJ, Muller C, et al. (2010) Meta-analysis: effect of B-type natriuretic peptide testing on clinical outcomes in patients with acute dyspnea in the emergency setting. Annals of Internal Medicine 153(11): 728–735.
8c.	Madsen MV, Gøtzsche PC, Hrobjartsson A (2009) Acupuncture treatment for pain: systematic review of randomised clinical trials with acupuncture, placebo acupuncture, and no acupuncture groups. BMJ 338: a3115. DOI:10.1136/bmj.a3115.
9a.	Santangeli P, Di Biase L, Dello Russo A, Casella M, Bartoletti S et al (2010) Meta-analysis: age and effectiveness of prophylactic implantable cardioverter-defibrillators. Annals of Internal Medicine 153(9): 592–599.
9b.	Ceglia L, Lau J, Pittas AG (2006) Meta-analysis: efficacy and safety of inhaled insulin therapy in adults with diabetes mellitus. Annals of Internal Medicine 145(9): 665–675.
9c.	Lin J S, O'Connor E, Whitlock EP, Beil TL (2010) Behavioral counseling to promote physical activity and a healthful diet to prevent cardiovascular disease in adults: a systematic review for the U.S. Preventive Services Task Force. Annals of Internal Medicine 53(11): 736–750.
9d.	Zoungas S, Ninomiya T, Huxley R, Cass A, Jardine M, et al. (2009) Systematic review: sodium bicarbonate treatment regimens for the prevention of contrast-induced nephropathy. Annals of Internal Medicine 151: 631–638.
10a.	Chandra D, Parasini E, Mozaffarian D (2009) Meta-analysis: travel and risk for venous thromboembolism. Annals of Internal Medicine 151(3): 180–190.
10b.	Thomson H, Thomas S, Sellstrom E, Petticrew M (2009) The health impacts of housing improvement: a systematic review of intervention studies from 1887 to 2007. American Journal of Public Health 99 Suppl 3: S681–S692.
10c.	Wells G, Parkash R, Healey JS, Talajic M, Arnold M, Sullivan S, et al. (2011) Cardiac resynchronization therapy: a meta-analysis of randomized controlled trials. CMAJ 183(4): 421–429. DOI:10.1503/cmaj.101685.
11a.	Levy G, Hill MJ, Ramirez CI, Correa L, Ryan ME, et al. (2012) The use of follicle flushing during oocyte retrieval in assisted reproductive technologies: a systematic review and meta-analysis. Human Reproduction 27(8): 2373–2379.
11b.	McKnight RF, Adida M, Budge K, Stockton S, Goodwin GM, et al. (2012) Lithium toxicity profile: a systematic review and meta-analysis. Lancet 379(9817): 721–728. DOI:10.1016/S0140-6736(11)61516-X.
12a.	Timmons BW, Leblanc AG, Carson V, Connor Gorber S, Dillman C, et al. (2012) Systematic review of physical activity and health in the early years (aged 0–4 years). Applied Physiology, Nutrition and Metabolism 37(4): 773–792.
12b.	Stradling C, Chen YF, Russell T, Connock M, Thomas GN, et al. (2012) The effects of dietary intervention on HIV dyslipidaemia: a systematic review and meta-analysis. PLoS ONE 7(6):e38121. Epub 2012 Jun 11.

### Section 1: TITLE

**Item 1: Title.**

**Identify the report as a systematic review, meta-analysis, or both.**

Examples: **1a.** “Systematic review and meta-analysis of the diagnostic and therapeutic role of water-soluble contrast agent in adhesive small bowel obstruction.”

**1b.** “Inhaled corticosteroids vs placebo for preventing COPD [chronic obstructive pulmonary disease] exacerbations: a systematic review and metaregression of randomized controlled trials.”

Explanation: The abstract should make it clear that the report is a systematic review, meta-analysis, or both (examples 1a and 1b). Search filters have been developed to identify systematic reviews [16], but inclusion of the words “systematic review” or “meta-analysis” in the title may improve indexing and electronic searching.

We also suggest using informative titles that incorporate the PICOS approach (participants, interventions, comparators, outcomes, and study designs). This provides key information about the scope of the systematic review. As including all elements of the PICOS approach may make the title unwieldy, we suggest including the most important of these elements in the title. These might be the elements that make this review unusual, or that assist readers in searching for the review.

### Section 2: BACKGROUND

**Item 2: Objectives.**

**The research question including components such as participants, interventions, comparators, and outcomes.**

Examples: **2a.** “To assess the effect on survival of supportive care and chemotherapy versus supportive care alone in advanced NSCLC [non-small cell lung cancer].”

**2b.** “To evaluate the risk of serious asthma-related events among patients treated with formoterol.”

**2c.** “The objective of this study was to investigate the predictive value of C-reactive protein in critically ill patients.”

Explanation: Irrespective of the strength and nature of the results reported in the abstract, readers should be able to assess the questions that the review intended to address. The objectives in an abstract should convey succinctly the broad aims of the systematic review. Objectives should reflect what the review intended to evaluate, such as benefit (example 2a), harms (example 2b), association, predictive value (example 2c), of the intervention or exposure of interest and the population or context in which this is being studied.

### Section 3: METHODS

**Item 3: Eligibility criteria.**

**Study and report characteristics used as criteria for inclusion.**

Examples – study characteristics: **3a.** “We included randomised controlled trials testing the combination of long-acting ß_2_- agonists in combination with inhaled corticosteroids (ICS) versus the same or an increased dose of ICS for a minimum of at least 28 days in children and adolescents with asthma.”

**3b.** “… randomized trials of compression stockings versus no stockings in passengers on flights lasting at least four hours. Trials in which passengers wore a stocking on one leg but not the other, or those comparing stockings and another intervention were also eligible.”

Examples – report characteristics: **3c.** “… studies published in English, French, Spanish, Italian and German between 1966 and July, 2008 [were included].”

**3d.** “We performed a literature search of trials using MEDLINE (January 1966–December 2001) … we retrieved English- and non-English-language articles for review… we searched for both published and unpublished trials…”

Explanation: One of the key features distinguishing a systematic review from a narrative review is the pre-specification of eligibility criteria for including and excluding studies. A clear description of these allows the readers to assess the applicability of the systematic review findings [11]. Study eligibility characteristics are likely to include the study questions (PICOS)—types of participants included in the studies (often based on a common clinical diagnosis), the intervention of prime interest and possibly the specific comparison intervention, the main outcome(s) being assessed—and acceptable study designs (examples 3a and 3b).

Eligibility criteria for reports may also include the language of publication, the publication status (e.g., whether to include unpublished materials and abstracts) and the year of publication (example 3d). This is important as inclusion, or not, of studies published in languages other than English (examples 3c and 3d), unpublished data, or older data can influence the estimates of effect or association in meta-analyses [17],[18].

**Item 4: Information sources.**

**Key databases searched and date of last search.**

Examples: **4a.** “PubMed, ERIC and Cochrane Reviews databases from January 1980 to November 2007 were searched for studies…”

**4b.** “We searched MEDLINE, EMBASE, the Cochrane Central Register of Controlled Trials (CENTRAL), other trial registries and product information sheets through June 2008.”

Explanation: The abstract should briefly indicate how thorough and up-to-date the search was by listing key databases searched, and the date range (example 4a) or date of last search (example 4b). We recommend that if there are three or fewer databases, list them all; otherwise list the three that provided the majority of included studies.

**Item 5: Risk of bias assessment.**

**Methods for assessing risk of bias.**

Example: **5a.** “Risk of bias was assessed regarding randomisation, allocation sequence concealment, blinding, incomplete outcome data, selective outcome reporting, and other biases.”

Explanation: Problems in the design and conduct of individual studies can raise questions about the validity of their findings [19]. For example, reports of randomised trials with inadequate allocation sequence concealment are more likely to show exaggerated treatment effects [20]. And non-blinded assessors of subjective outcomes generate substantially biased effect estimates [21],[22]. It is therefore an important part of a systematic review to assess the validity of individual studies, and the risk that they will overestimate the true intervention effect. Authors should describe any methods they used to assess the risk of bias in the included studies (example 5a).

Many tools exist for assessing the overall risk of bias in included studies, including scales, checklists and individual components [23]. Most tools are scales in which various components of quality are scored and combined to give a summary score. This approach can be seriously misleading, however, and should be discouraged. A preferred approach requires authors to specify which individual methodological components they will assess and to provide a description and judgment for each component for each of the studies assessed [19]. For randomised trials, common components include: appropriate generation of the allocation sequence [24], concealment of the allocation sequence [20], blinding of participant and health care providers, blinding of outcome assessors [22], assessment of incomplete outcome data [25], and selective outcome reporting [26].

### Section 4: RESULTS

**Item 6: Included studies.**

**Number and type of included studies and participants, and relevant characteristics of studies.**

Examples: **6a.** “We included 22 trials involving 101 507 participants: 11 trials reported on presumptive pneumococcal pneumonia, 19 on all-cause pneumonia and 12 on all-cause mortality. The current 23-valent vaccine was used in 8 trials.”

**6b.** “Eight studies included in this review (n = 586 patients, median PEDro score = 8.0/10) evaluated various parameters, including the duration of patients' symptoms (0–12 months), duty cycle (20% and 100%), intensity (0.1–2.0 W/cm2), treatment time per session (4.5–15.8 minutes), number of treatments (6–39), and total energy applied per treatment (181–8,152 J).”

Explanation: The number of studies, number of participants, and characteristics of the included studies (examples 6a and 6b) enable readers to gauge the validity and applicability of the systematic review's results. These characteristics might include descriptors of the participants (e.g., age, severity of disease), range of interventions used (e.g., dose and frequency of drug administration), and measurement of outcomes (e.g., follow-up times).

**Item 7: Synthesis of results.**

**Results for main outcomes (benefits and harms), preferably indicating the number of studies and participants for each. If meta-analysis was done, include summary measures and confidence intervals.**

Examples: **7a.** “… CRT [cardiac resynchonization therapy] reduced all-cause mortality (6 trials, 4572 participants; risk ratio [RR], 0.83 [95% CI, 0.72 to 0.96]) and heart failure hospitalizations (4 trials, 4349 participants; RR, 0.71 [CI, 0.57 to 0.87]) without improving functional outcomes or quality of life.”

**7b.** “Six studies reported on maternal mortality and our meta-analysis showed a non-significant reduction (three randomised trials, relative risk 0.79, 0.53 to 1.05, P = 0.12; three non-randomised studies, 0.80, 0.44 to 1.15, P = 0.26).”

**7c.** “Eight studies presented adjusted odds ratios, ranging from 0.3 to 0.9, suggesting a reduced likelihood of self-reported sharing of non-N/S [non-needle/syringe] injecting paraphernalia associated with use of NSP [needle and syringe exchange programmes] or SIF [safer injection facilities].”

Explanation: The results for the main outcomes should be given in the abstract. If meta-analyses have been done, include for each the summary measure (estimated effect) and confidence interval. If the intention had been to perform meta-analysis, but no meta-analysis was done for one or more main outcomes, the reasons should be stated (e.g., heterogeneity too great).

The abstract should make clear the protocol-defined, pre-specified importance of each outcome reported, and should not report only those outcomes that have statistically significant or clinically important results.

Where possible, given space limitations, the number of studies and participants for each main outcome should be stated, particularly if only a small proportion of the total number of studies or patients in the systematic review contributed information on a particular outcome.

If there are no summary measures, some numerical data may still be given (example 7c), although authors should be wary of making this in the form of “vote counting” where the number of “positive” and “negative” studies is given. Vote counting takes no account of weighting of studies according to the amount of information they contain [27].

**Item 8: Description of effect.**

**Direction of the effect (i.e., which group is favoured) and size of the effect in terms meaningful to patients and clinicians.**

Examples: **8a.** “Radial access reduced major bleeding by 73% compared to femoral access (0.05% vs 2.3%, OR 0.27 [95% CI 0.16, 0.45], P<0.001).”

**8b.** “Length of hospital and critical care unit stay were both modestly reduced in the tested group compared with the control group, with a mean difference of −1.22 day (CI, −2.31 to −0.14 day) and −0.56 day (CI, −1.06 to −0.05 day), respectively.”

**8c.** “A small difference was found between acupuncture and placebo acupuncture: standardised mean difference −0.17 (95% confidence interval −0.26 to −0.08)… [in favour of acupuncture]…, corresponding to 4 mm (2 mm to 6 mm) on a 100 mm visual analogue scale.”

Explanation: The results should summarise the main outcomes in words and numbers. The wording should indicate the direction of the effect (e.g., lower, fewer, reduced; greater, more, increased) and the size of the effect using familiar units such as percentages, days, or kilograms. Example 8a makes clear the size of the effect even for readers who have difficulty interpreting relative risks and confidence intervals. When a percentage is used, the baseline risk should also be shown, which allows the reader to see what the absolute benefit or harm is, and calculate whichever measures they choose (example 8a). Authors should take care to make it clear whether the reported measure is an absolute or a relative one (e.g., where percentage is used as the units of measurement). Where possible, continuous outcome measures should be expressed in familiar units (example 8b), particularly when the standardised mean difference is used (example 8c).

### Section 5: DISCUSSION

**Item 9: Strengths and limitations of evidence.**

**Brief summary of strength and limitations of evidence (e.g., inconsistency, imprecision, indirectness, or risk of bias, other supporting or conflicting evidence).**

Examples: **9a.** “Four potentially eligible trials were not included in the meta-analysis because mortality data by age group were not available.”

**9b.** “All trials were open label, which may introduce bias. Most of the trials were of 24 weeks' duration or less, limiting assessment of long-term safety.”

**9c.** “Meta-analyses for some outcomes had large statistical heterogeneity or evidence for publication bias. Only 11 trials followed outcomes beyond 12 months.”

**9d.** “Meta-regression showed that small, poor-quality studies that assessed outcomes soon after radiocontrast administration were more likely to suggest benefit (P<0.05 for all).”

Explanation: The abstract should briefly describe the strengths and limitations of the evidence across studies [28]. Limitations may include: risk of bias common to many or all studies, such as lack of blinding for subjective outcomes (example 9b) or unavailability of data (example 9a); inconsistency of effect or association, as demonstrated by high heterogeneity (examples 9c and 9d); imprecision, e.g., due to few events or small sample sizes; indirectness of the evidence, such as the use of an intermediate or short-term outcome (examples 9b and 9c); and likely publication bias (example 9c). Potential strengths of the overall body of evidence that might apply for a particular outcome of a systematic review include: a large effect (example 8a); demonstration of a dose-response relationship (example 10a, below); and that all biases would be likely to reduce the effect rather than increase it. One or more of these strengths and limitations may apply to each of the outcomes of the systematic review being described in the abstract. Some of this information may be combined with item 6, above, when describing the included studies, however a summary of the overall strengths and limitations of the evidence might also be helpful.

**Item 10: Interpretation.**

**General interpretation of the results and important implications.**

Examples: **10a.** “Travel is associated with a nearly 3-fold higher risk for VTE [venous thromoboembolism], with a dose-response relationship of 18% higher risk for each 2-hour increase in travel duration.”

**10b.** “Housing improvements, especially warmth improvements, can generate health improvements; there is little evidence of detrimental health impacts. The potential for health benefits may depend on baseline housing conditions and careful targeting of the intervention. Investigation of socioeconomic impacts associated with housing improvement is needed to investigate the potential for longer-term health impacts.”

**10c.** “The cumulative evidence is now conclusive that the addition of cardiac resynchronization to optimal medical therapy or defibrillator therapy significantly reduces mortality among patients with heart failure.”

Explanation: Remembering that some readers may struggle with interpreting the statistical results, an overall summary of the main effects—positive or negative—should be given (example 10a). This could include an indication of what is clear (example 10c), what important uncertainties remain (example 10b), and whether there is ongoing research addressing these.

If there is insufficient evidence from well-conducted studies to answer the review's question, this should be made clear to the reader. When the results are not statistically significant, authors should distinguish between those where there is insufficient evidence to rule out a difference between treatments (wide confidence interval), and those which have sufficient evidence that an important difference is unlikely (narrow confidence interval).

If the conclusions of the review differ substantially from previous systematic reviews, then some explanation might also be provided. Reference could be made to known ongoing studies that have the potential to change the result of the review. Possible implications for policy and practice should be stated.

### Section 6: OTHER

**Item 11: Funding.**

**Primary source of funding for the review.**

Examples: **11a.** “This work was supported, in part, by the Program in Reproductive and Adult Endocrinology, NICHD, NIH, Bethesda, MD. The authors have no competing interests to declare.”

**11b.** “Funding: National Institute for Health Research Programme Grant for Applied Research.”

Explanation: Studies of the relationship between pharmaceutical company funding and results of clinical trials have shown that sponsored studies are more likely to have outcomes favouring the sponsor [29],[30]. This is also the case for systematic reviews [31]. Therefore, the abstract should indicate whether the sponsor of the research or the researchers might have a conflict of interest in respect of the findings of the systematic review, for example, as the manufacturer of the intervention being evaluated (examples 11a and 11b). The abstract should include the main source of funding for the systematic review, whether from host institutions or from external bodies.

**Item 12: Registration.**

**Registration number and registry name.**

Examples: **12a.** “PROSPERO registration: CRD42011001243.”

**12b.** “PROSPERO 2011:CRD42011001329.”

Explanation: Registration of systematic reviews provides a record of reviews that have been initiated, even if they have not been published. It is therefore a means of alerting researchers to systematic reviews that are in progress, and serves as a public record of the proposed systematic review. It also helps to detect reporting bias by enabling better identification of unpublished systematic reviews, and also to compare the methods or outcomes reported in published reviews with those originally proposed in registered protocols [32]. The abstract should record the name of the database with which the review is registered, and the registration number. Cochrane reviews are an exception to this requirement, as they are preceded by a peer reviewed protocol that is published in the Cochrane Library and can be downloaded from there.

## Discussion

The title of a systematic review is its first signal of its relevance to potential readers. Few titles will entice a reader to invest additional time, but when they do, they ordinarily start—and quite often end—with the abstract. The first impression is therefore crucial.

We strongly recommend the use of structured abstracts for reporting systematic reviews, as does the PRISMA Statement [11]. We recognise that journals have developed their own set of headings that are considered appropriate for reporting systematic reviews, and it is not our intention to suggest changes to these headings, but to recommend what should be reported under them. The order of items and the headings are therefore flexible. For example, the strengths and limitations may be stated at the end of the Results, under a separate heading, or with the Discussion or Conclusions, depending on journal requirements. It may also be possible to combine items from the checklist into one sentence. For example, limitations may be combined with a description of the included studies (i.e., items 6 and 9 from the checklist).

We have suggested reporting a minimum set of items. We do not advocate that abstracts replace full articles in informing decision making, but we recognise that for many time-pressed readers, or for those with limited access to the full texts of reports, it is important that abstracts contain as much information as is feasible within the word limit of abstracts. Indeed, for readers who do not understand the language of publication of the article, the translated abstract may have far more relevance than the full-text article.

A checklist is not sufficient to ensure good abstract writing. For example, the abstract should clearly and truthfully reflect the full report, and not selectively report results that are statistically significant while not referring to those that were not. Similarly, the abstract should only draw conclusions that are substantiated by data from the full report and analyzed as described in the protocol, rather than selectively emphasising interesting results that were a minor or *ad hoc* component of the analysis. In brief, the abstract should be an unbiased representation of the full report. We also suggest that peer and editorial review processes related to the abstract should explicitly check this.

A particularly difficult area is the Discussion section of an abstract. The checklist includes two items with several elements. We suggest that authors let the reader know whether they feel their question has been answered, or whether there is still uncertainty before presenting practice and policy implications. These statements should be clearly backed by the results given in the abstract, and by presentation of the strengths and limitations of the evidence in the review.

We encourage journals and conference organisers to endorse the use of PRISMA for Abstracts, in a similar way to CONSORT for Abstracts [33]. This may be done by modifying their instructions to authors and including a link to the checklist on their website. It has been demonstrated that the number of checklist items reported is improved in journals that require checklist completion as part of the submission process [34].

Abstracts should not replace full articles in informing decision making, but for time-pressed readers and those with limited access to full text reports, the abstract must stand alone in presenting a clear and truthful account of the research. The PRISMA for Abstracts checklist will guide authors in presenting an abstract that facilitates a quick assessment of review validity, an explicit summary of results, facilitates pre-publication or conference selection peer review, and enables efficient perusal of electronic search results.

## Abbreviations

PICOS: participants, interventions, comparators, outcomes, and study designs

## References

[1] Bastian H, Glasziou P, Chalmers I (2010) Seventy-five trials and eleven systematic reviews a day: how will we ever keep up? PLoS Med 7 (9) e1000326 doi:10.1371/journal.pmed.1000326. 20877712 10.1371/journal.pmed.1000326 PMC2943439

[2] Dogan RI, Murray GC, Neveol A, Lu Z (2009) Understanding PubMed user search behavior through log analysis. Database: Article ID bap018. doi:10.1093/database/bap018. 10.1093/database/bap018 PMC2797455 20157491

[3] Tovey D (2010) Impact of Cochrane reviews. Cochrane Database Syst Rev 7 (8) ED000007. 10.1002/14651858.ED000007 PMC10846555 21833930

[4] Ad Hoc Working Group for Critical Appraisal of the Medical Literature (1987) A proposal for more informative abstracts of clinical articles. Ann Intern Med 106: 598–604. 3826959

[5] Haynes RB, Mulrow CD, Huth EJ, Altman DG, Gardner MJ (1990) More informative abstracts revisited. Ann Intern Med 113: 69–76. 2190518 10.7326/0003-4819-113-1-69

[6] Hartley J (2000) Clarifying the abstracts of systematic literature reviews. Bull Med Libr Assoc 88 (4) 332–337. 11055300 PMC35254

[7] Froom P, Froom J (1993) Deficiencies in structured medical abstracts. J Clin Epidemiol 46 (7) 591–594. 8326342 10.1016/0895-4356(93)90029-z

[8] Hopewell S, Eisinga A, Clarke M (2008) Better reporting of randomized trials in biomedical journal and conference abstracts. J Info Sci 34 (2) 162–173.

[9] Hopewell S, Clarke M, Askie L (2006) Reporting of trials presented in conference abstracts needs to be improved. J Clin Epidemiol 59: 681–684. 16765270 10.1016/j.jclinepi.2005.09.016

[10] Beller EM, Glasziou PP, Hopewell S, Altman DG (2011) Reporting of effect direction and size in abstracts of systematic reviews. JAMA 306 (18) 1981–1982. 22068989 10.1001/jama.2011.1620

[11] Liberati A, Altman DG, Tetzlaff J, Mulrow C, Gotzsche PC, et al (2009) The PRISMA statement for reporting systematic reviews and meta-analyses of studies that evaluate health care interventions: explanation and elaboration. PLoS Med 6 (7) e1000100 doi:10.1371/journal.pmed.1000100. 19621070 10.1371/journal.pmed.1000100 PMC2707010

[12] Sacks HS, Berrier J, Reitman D, Ancona-Berk VA, Chalmers TC (1987) Meta-analysis of randomized controlled trials. New Engl J Med 316: 450–455. 3807986 10.1056/NEJM198702193160806

[13] Murphy MK, Black NA, Lamping DL, McKee CM, Sanderson CFB, et al. (1998) Consensus development methods, and their use in clinical guideline development. Health Technol Assessment 2 (3). 9561895

[14] Moher D, Hopewell S, Schulz KF, Montori V, Gotzche PC, et al (2010) CONSORT 2010 explanation and elaboration: updated guidelines for reporting parallel group randomised trials. BMJ 340: c869 doi:10.1136/bmj.c869. 20332511 10.1136/bmj.c869 PMC2844943

[15] Hopewell S, Clarke M, Moher D, Wager E, Middleton P, et al (2008) CONSORT for reporting randomized controlled trials in journal and conference abstracts: explanation and elaboration. PLoS Med 5 (1) e20 doi:10.1371/journal.pmed.0050020. 18215107 10.1371/journal.pmed.0050020 PMC2211558

[16] Montori VM, Wilczynski NL, Morgan D, Haynes RB (2005) Optimal search strategies for retrieving systematic reviews from Medline: analytical survey. BMJ 330: 68. 15619601 10.1136/bmj.38336.804167.47 PMC543864

[17] Egger M, Juni P, Bartlett C, Holenstein F, Sterne J (2003) How important are comprehensive literature searches and the assessment of trial quality in systematic reviews? Empirical study. Health Technol Assessment 7 (1) 1–76. 12583822

[18] Song F, Parekh S, Hooper L, Loke YK, Ryder J, et al (2010) Dissemination and publication of research findings: an updated review of related biases. Health Technol Assessment 14 (8) iii, ix–xi, 1–193. 10.3310/hta14080 20181324

[19] Higgins JPT, Altman DG, Gotzsche PC, Juni P, Moher D, et al (2011) The Cochrane Collaboration's tool for assessing risk of bias in randomised trials. BMJ 343: d5928 doi:10.1136/bmj.d5928. 22008217 10.1136/bmj.d5928 PMC3196245

[20] Pildal J, Hrobjartsson A, Jorgensen K, Hilden J, Altman D, et al (2007) Impact of allocation concealment on conclusions drawn from metaanalyses of randomized trials. Int J Epidemiol 36: 847–857. 17517809 10.1093/ije/dym087

[21] Schulz KF, Grimes DA (2006) The Lancet handbook of essential concepts in clinical research. London: Elsevier.

[22] Hrobjartsson A, Thomsen AS, Emanuelsson F, Tendal B, Hilden J, et al (2012) Observer bias in randomised clinical trials with binary outcomes: systematic review of trials with both blinded and non-blinded outcome assessors. BMJ 344: e1119 doi:10.1136/bmj.e1119. 22371859 10.1136/bmj.e1119

[23] Jüni P, Altman DG, Egger M (2001) Systematic reviews in health care: Assessing the quality of controlled clinical trials. BMJ 323: 42–46. 11440947 10.1136/bmj.323.7303.42 PMC1120670

[24] Als-Nielsen B, Gluud LL, Gluud C (2004) Methodological quality and treatment effects in randomized trials: a review of six empirical studies. 12th Cochrane Colloquium, Ottawa (Canada).

[25] Tierney JF, Stewart LA (2005) Investigating patient exclusion bias in meta-analysis. Int J Epidemiol 34: 79–87. 15561753 10.1093/ije/dyh300

[26] Chan AW, Hróbjartsson A, Haahr MT, Gøtzsche PC, Altman DG (2004) Empirical evidence for selective reporting of outcomes in randomized trials: comparison of protocols to published articles. JAMA 291: 2457–2465. 15161896 10.1001/jama.291.20.2457

[27] Guyatt G, Oxman AD, Akl EA, Kunz R, Vist G, et al (2011) GRADE guidelines: 1. Introduction – GRADE evidence profiles and summary of findings tables. J Clin Epidemiol 64: 383–394. 21195583 10.1016/j.jclinepi.2010.04.026

[28] Antman EM, Lau J, Kupelnick B, Mosteller F, Chalmers TC (1992) A comparison of results of meta-analyses of randomized control trials and recommendations of clinical experts: treatments for myocardial infarction. JAMA 268: 240–248. 1535110

[29] Als-Nielson B (2003) Association of funding and conclusions in randomized drug trials: a reflection of treatment effect or adverse events? JAMA 290 (7) 921–928. 12928469 10.1001/jama.290.7.921

[30] Lexchin J, Bero LA, Djulbegovic B, Clark O (2003) Pharmaceutical industry sponsorship and research outcome and quality: Systematic review. BMJ 326: 1167–1170. 12775614 10.1136/bmj.326.7400.1167 PMC156458

[31] Jørgensen AW, Hilden J, Gøtzsche PC (2006) Cochrane reviews compared with industry supported meta-analyses and other meta-analyses of the same drugs: systematic review. BMJ 333: 782–785. 17028106 10.1136/bmj.38973.444699.0B PMC1602036

[32] Booth A, Clarke M, Ghersi D, Moher D, Petticrew M, et al (2011) Establishing a minimum dataset for prospective registration of systematic reviews: an international consultation. PLoS ONE 6 (11) e27319 doi:10.1371/journal.pone.0027319. 22110625 10.1371/journal.pone.0027319 PMC3217945

[33] Hopewell S, Clarke M, Moher D, Wager E, Middleton P, et al (2008) CONSORT for reporting randomized controlled trials in journal and conference abstracts: explanation and elaboration. PLoS Med 5 (1) e20 doi:10.1371/journal.pmed.0050020. 18215107 10.1371/journal.pmed.0050020 PMC2211558

[34] Hopewell S, Ravaud P, Baron G, Boutron I (2012) Effect of editors' implementation of CONSORT guidelines on the reporting of abstracts in high impact medical journals: interrupted time series analysis. BMJ 344: e4178 doi:10.1136/bmj.e4178. 22730543 10.1136/bmj.e4178 PMC3382226

